# Design and synthesis of coumarin-based organoselenium as a new hit for myeloprotection and synergistic therapeutic efficacy in adjuvant therapy

**DOI:** 10.1038/s41598-018-19854-5

**Published:** 2018-02-01

**Authors:** Arup Ranjan Patra, Somnath Singha Roy, Abhishek Basu, Avishek Bhuniya, Arin Bhattacharjee, Subhadip Hajra, Ugir Hossain Sk, Rathindranath Baral, Sudin Bhattacharya

**Affiliations:** 1 Department of Cancer Chemoprevention, Chittaranjan National Cancer Institute, 37, S. P. Mukherjee Road, Kolkata, 700 026 West Bengal India; 2 Centre of Biomedical Research, Sanjay Gandhi Post-Graduate Institute of Medical Sciences Campus, Raebareli Road, Lucknow, 226014 India; 3 Department of Immunoregulation and Immunodiagnostics, Chittaranjan National Cancer Institute, 37, S. P. Mukherjee Road, Kolkata, 700 026 West Bengal India; 4 Natural Product Chemistry & Process Development Division, CSIR-Institute of Himalayan Bioresource Technology, Palampur, 176061 Himachal Pradesh India

## Abstract

A newly designed organoselenium compound, methyl substituted umbelliferone selenocyanate (MUS), was synthesized as a primary hit against the myelotoxic activity of carboplatin. MUS was administered at 6 mg/kg b.wt, p.o. in concomitant and pretreatment schedules with carboplatin (12 mg/kg b.wt, i.p. for 10 days) in female Swiss albino mouse. MUS treatment reduced (P < 0.001) the percentage of chromosomal aberrations, micronuclei formation, DNA damage and apoptosis in murine bone marrow cells and also enhanced (P < 0.001) the bone marrow cell proliferation of the carboplatin-treated mice. These activities cumulatively restored the viable bone marrow cell count towards normalcy. Myeloprotection by MUS was achieved, in part, due to a significant reduction in the ROS/RNS formation and restoration of glutathione redox pool. Additionally, MUS synergistically enhanced the cytotoxicity of carboplatin against two human cancer cell lines (MCF-7 and Colo-205). Furthermore, MUS can effectively potentiate the antitumour activity of carboplatin against two murine cancers (Dalton’s Lymphoma and Sarcoma-180) *in vivo*. These preclinical findings clearly indicate that MUS can improve the therapeutic index of carboplatin and ensures more effective therapeutic strategy against cancer for clinical development.

## Introduction

Despite much advancement in targeted and customized cancer chemotherapy, platinum-based drugs are in routine clinical use in low and middle-income countries. The second generation platinum salt, carboplatin (cis-diamine [1, 1-cyclobutanedicarboxylato]-platinum [II]) or CBDCA is generally used for the treatment of head and neck, lung and ovarian cancer^1^. Like other chemotherapeutic platinum drugs, CBDCA interacts with cellular DNA to form DNA-platinum adduct which generally crosslinks DNA^2^, resulting in cytotoxicity to cancer as well as normal cells. Hence, the substantial therapeutic benefit of CBDCA treatment is compensated by various clinical complications like cardiotoxicity^3^, myelotoxicity^4,5^, ototoxicity^6^ and nephrotoxicity^7^. Myelotoxicity induced by CBDCA limits its therapeutic dose^4,5^ because of its unwanted toxicity in highly proliferative organs like bone marrow. CBDCA induces substantial DNA crosslinking. This leads to increases in replication error and eventually generates mutation. In this scenario, protection from CBDCA-induced myelotoxicity without compromising its chemotherapeutic efficacy is a clinical necessity.

The concurrent use of nontoxic chemoprotective agent offers effective management of chemotherapy-associated toxicity^8^. In a recently developed approach, selenium is considered as a modulator of chemotherapeutic outcome as it can attenuate the chemotherapy-induced toxicity and stimulates the antineoplastic efficacy^9^. Some biochemical properties of selenium like its (a) incorporation into proteins as selenocysteine and selenomethionine during translation, (b) presence of the functional moiety of 25 selenoenzyme in human^10,11^ and (c) dual behavior to act as antioxidant and pro-oxidant depending upon cellular redox environment make it unique among other possible candidates. In addition, this micronutrient is essential for bone homeostasis^12^ and it protects bone from various diseases like rheumatoid arthritis, osteoarthritis, and osteoporosis^13^. Thus the efficacy of this trace element in the protection of normal cells, tissues and organs from mutagens can be capitalized to achieve a better therapeutic outcome through the possible use of chemotherapy in higher doses, longer duration or both. In post-SELECT (Selenium and Vitamin E Cancer Prevention Trial) era, attention is now shifted towards various organic selenium compounds due to their better bioavailability and safety profile^14–16^ than inorganic selenium compounds.

In the present work, a coumarin-based organoselenium compound, methyl substituted umbelliferone selenocyanate or MUS, was designed and synthesized as a potential hit against carboplatin-induced myelotoxicity. Coumarin and its analogues are well known for their various biological activities including anti-steroid^17^, anticancer^18^ and many others^19–21^. Moreover, the nontoxic character of the coumarin-based compounds also motivated us to use this bioactive scaffold^22^ to synthesize a new coumarin-based organoselenium compound. The oral LD_50_ and effective dose of MUS were determined in female Swiss albino mice according to OECD guidelines. MUS at its most effective dose were evaluated for its chemoprotective potential against CBDCA-induced myelotoxicity in bone marrow cells of Swiss albino mouse. Therapeutic effects of MUS alone or in combination with CBDCA were also investigated *in vitro* and *in vivo* as well. *In vitro* therapeutic efficacy was evaluated against MCF-7 and Colo-205 whereas *in vivo* study was carried out in Swiss albino mice bearing Dalton’s Lymphoma or Sarcoma-180 in solid as well as ascites tumour forms.

## Results

### LD_50_ of MUS

The oral LD_50_ of the MUS was found to be >2000 mg/kg b.wt. (based on an assumed sigma of 0.5). No sign of toxicity or moribund state were observed among the live animals during the entire duration of the study.

### Dose selection of MUS

**Figure 1:**
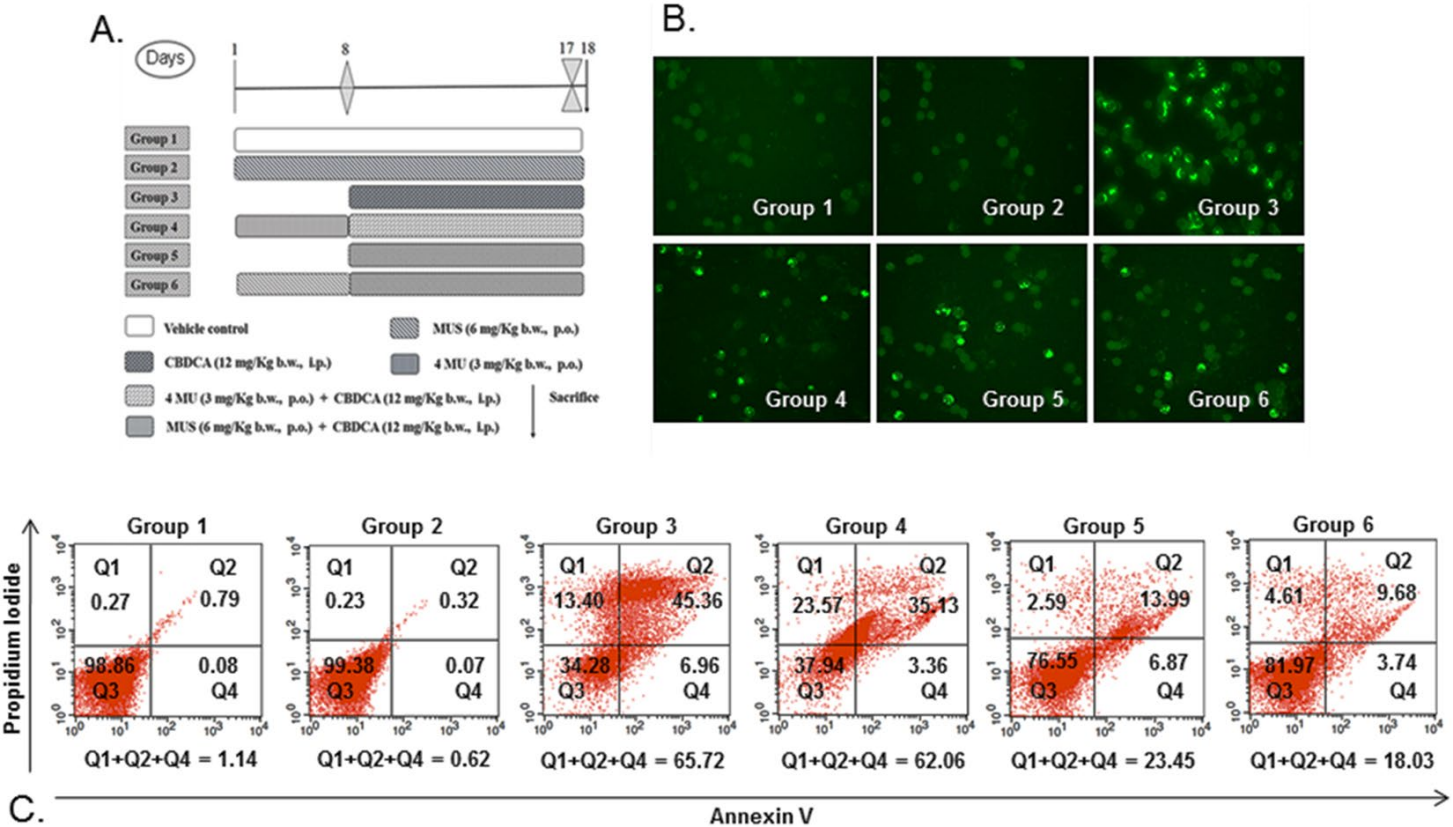
(**A**) Graphical presentation of different groups of mice for *in vivo* chemoprotection study. (**B**) Effect of MUS on CBDCA-induced cytotoxicity and clastogenic effect in murine bone marrow cells. Photomicrographs after TUNEL assay were taken at 200× magnification. Brightly-stained nucleus represents an apoptotic cell, whereas, unstained cells represent non-apoptotic cells. (**C**) Inhibition of CBDCA-induced cell death of murine bone marrow cells by MUS. After treatment completion, murine bone marrow cells were isolated and stained with annexin-V and PI as described in Methods and analyzed by flow cytometry. The figure is a representative profile of at least three experiments in duplicate.

Optimized oral dose of MUS was selected through the evaluation of some clinical safety (Suppl Table 1 and Suppl Table 2) and efficacy (Suppl Table 3) endpoints. A comparative study of three different oral doses of MUS, 3 mg/Kg b.wt., 6 mg/Kg b.wt. and 12 mg/Kg b.wt., were carried out on Swiss albino mice. MUS at the oral dose of 3 mg/Kg b.wt. and 6 mg/Kg b.wt. showed no clinically detectable toxicity. The safety of MUS at these two doses was reflected by the gain in body weight (P < 0.05); increase in Hb level (P < 0.05) and RBC count, compared to vehicle control. At 12 mg/Kg b.wt, however, MUS slightly enhanced the LPO levels in liver and ALT, AST, BUN and creatinine level as well. Consequently, 12 mg/Kg b.wt. dose was ruled out from further study. Interestingly, WBC count was increased (P > 0.05) in all the three dose groups, with respect to vehicle control which suggested the immunomodulatory property of selenium. Increase in cell number in the femoral bone marrow, spleen and thymus were observed in all three dose groups but no visible abnormalities were noticed in these organs during necroscopy. The proportion of monocytes, eosinophil, and basophil were found negligible in all these three dose groups indicating no hypersensitivity after MUS administration. Antioxidative defence system was strengthened by administration of MUS at 3 mg/Kg b.wt. and 6 mg/Kg b.wt. Among these two dose group, significant increase in GSH level (P < 0.05) and activities of GST (P < 0.05), GPx (P < 0.05), SOD (P < 0.05) and Catalase (P < 0.05) were observed at the dose of 6 mg/Kg b.wt. of MUS. Considering all the above results, 6 mg/Kg b.wt. of the oral dose was evaluated in the in vivo chemoprotection and therapeutic efficacy study. Experimental groups for chemoprotection study were designed according to Fig. 1A.

### MUS significantly reduced apoptosis in bone marrow cells

TUNEL assay was used to estimate the extent of apoptosis in bone marrow cells (Fig. 1B and Table 1). Apoptotic index (AI) in Group 1 (vehicle treatment) and Group 2 (MUS only) was 0.91% and 0.47%, respectively. In Group 3 animals, CBDCA administration significantly (P < 0.001) increased the AI to 53.14% in comparison to vehicle control. MUS treatment significantly (P < 0.001) lowered the AI to 21.46% and 16.93%, respectively, in concomitant treatment and pretreatment schedules, respectively, in comparison with Group 3. 4MU, the organic scaffold of the MUS without selenium, was able to reduce the AI to 49.32% (P > 0.05) in comparison to Group 3.

**Table 1:** Alteration of chromosomal aberration, micronuclei formation, DNA damage (comet assay), BrdU labelling index and apoptotic index (AI) in bone marrow cells by MUS in CBDCA-treated mice.

Groups	Chromosomal aberrations (CA)		Micronuclei frequency (MN)		DNA damage (comet assay)			BrdU labelling index (BrdU LI)	Apoptotic index (AI)
	% of aberrated cells	% of inhibition	% of micronucleated cells	% of inhibition	Damaged cells (%)	Comet tail length (μm)	Olive tail moment		
Group 1	7.2 ± 0.6	-------	0.2 ± 0.02	-------	9.7 ± 0.8	14.1 ± 0.7	2.6 ± 0.7	54.1 ± 4.32	0.91 ± 0.07
Group 2	7.1 ± 0.8	-------	0.2 ± 0.01	-------	8.2 ± 0.8	8.9 ± 1.3^*^	1.2 ± 0.6^*^	55.0 ± 4.4	0.47 ± 0.09
Group 3	41.2 ± 3.4^*^	--------	1.7 ± 0.07^*^	--------	48.3 ± 3.9^*^	31.2 ± 2.3^*^	7.3 ± 0.4^*^	23.8 ± 1.9^*^	53.14 ± 5.1^*^
Group 4	32.1 ± 2.9^*^	22.0	0.9 ± 0.08^*^	46.8	37.1 ± 2.1^*^	26.1 ± 0.9^*^	4.2 ± 0.6^*^	29.7 ± 2.1	49.32 ± 4.7
Group 5	24.3 ± 2.5^*^	40.9	0.5 ± 0.06^*^	67.6	16.4 ± 1.2^*^	16.9 ± 0.4^*^	3.4 ± 0.7^*^	34.6 ± 3.5^*^	21.46 ± 2.8^#^
Group 6	18.6 ± 2.7^*^	54.8	0.4 ± 0.03^*^	76.3	12.4 ± 0.6^*^	17.8 ± 1.2^*^	3.1 ± 0.5^*^	41.3 ± 3.8^*^	16.93 ± 2.1^*^

Data are represented as mean ± SD, n = 6. *Denotes (P < 0.001), ^#^denotes (P < 0.01) as compared to their respective control groups. Comparisons were made between Group 1 with Group 2 and 3; Group 3 with Group 4, 5 and 6.

Apoptosis in bone marrow cells was also determined by annexin V/PI staining followed by flow cytometric analysis (Fig. 1C). Dead cell population in Group 1 (vehicle treatment) and Group 2 (MUS only) was found to be 1.14% and 0.62%. This was significantly (P < 0.001) increased up to 65.72% in Group 3 animals due to CBDCA administration. MUS treatment significantly (P < 0.001) lowered the CBDCA-induced cell death to 23.45% and 18.03% in concomitant treatment (Group 5) and pretreatment (Group 6) schedules respectively compared with the CBDCA control group (Group 3). 4MU treatment (Group 4) could not provide any protection to bone marrow cells as the cell death found in this group was 62.06% (P > 0.05).

### MUS modulate bone marrow cell proliferation and chromosomal aberration

**Figure 2:**
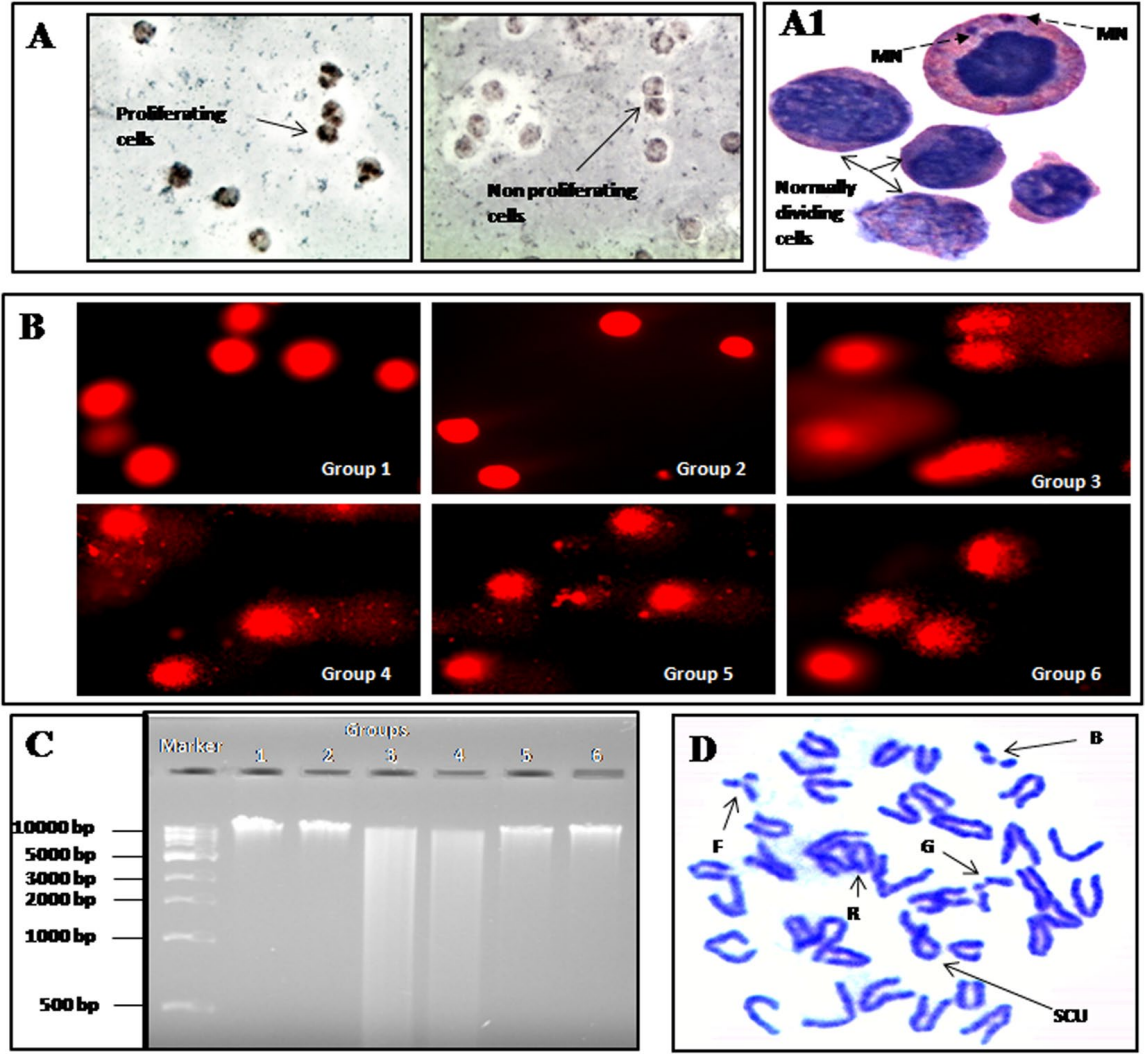
Effect of MUS on CBDCA-induced cytotoxicity and clastogenic effect in murine bone marrow cells. (**A**) Representative photomicrograph (taken at 400 × magnification) of cell proliferation, where proliferating cells are BCIP/NBT stained and non-proliferating cells are unstained. (**A1**) Representative photomicrograph for the formation of micronuclei (indicated by broken arrow) after CBDCA treatment (1000X). (**B**) Effect of MUS on CBDCA-induced DNA damage in murine bone marrow cells. Photomicrographs after comet assay were taken at 400 × magnification. (**C**) Photomicrograph of agarose gel electrophoresis of genomic DNA. (**D**) Representative photomicrograph of chromosomal aberrations in a single metaphase plate taken at 1000 × magnification. Different anomalies like break [B], sister chromatid union [SCU], fragmentation [F], gap [G] and ring [R] are indicated.

Viable femoral bone marrow cells in the hosts were counted by trypan blue dye exclusion method. The proliferation of cells in the bone marrow niche was determined by BrdU labelling method and expressed by BrdU labelling index (BrdU LI; Fig. 2A and Table 1). Viable cell count in Group 1 (vehicle treatment) and Group 2 (MUS only) was found to be (9.76 ± 1.08) × 10^6^ and (11.29 ± 1.17) × 10^6^/femur, respectively, whereas the BrdU LI was found to be 54.17% and 55.01%, respectively. In Group 3 animals, CBDCA administration resulted in significant (P < 0.001) reduction of the BrdU LI to 23.82% which, in turn, lowered the viable bone marrow cell count by 43.54% in comparison to Group 1. MUS administration in concomitant treatment schedule (Group 5) increased the BrdU LI to 34.62% (P < 0.001) and the viable cell count by 48.45% (P < 0.01) with respect to Group 3. Pretreatment by MUS (Group 6) increased the BrdU LI to 41.39% (P < 0.001) and the viable cell count by 69.14% (P < 0.001) [(9.32 ± 1.29) × 10^6^/femur] with respect to Group 3. 4MU treatment increased the BrdU LI to 29.74% (P > 0.05) and the viable cell count by 3.99% (P > 0.05) [(5.73 ± 1.04) × 10^6^/femur] in comparison with Group 3. On the other hand, mice treated with CBDCA showed significant (P < 0.001) a high proportion of chromosomal aberrations (CA) (Fig. 2D and Table 1) in bone marrow cells which were inhibited significantly (P < 0.001) by 40.95% and 54.88% in concomitant and pretreatment schedule respectively. Administration of 4 MU also inhibited the CA formation by 22.09%. Categorization of various structural aberrations in different groups is provided in supplementary data (Suppl Table 4). These results clearly demonstrated the bone marrow cell survival promotion by MUS. Hence it is clear that the positive modulation of proliferation and survival resulted in the higher viability of bone marrow cells.

### Protection of DNA damage and genomic DNA integrity by MUS

DNA damage in femoral bone marrow cells was evaluated by comet assay (Fig. 2B and Table 1) and damaged cell population (%), average tail length (μm) and Olive tail moment were analyzed. Large round head with no tail was observed in bone marrow cells of untreated vehicle-treated mice (Group 1), as well as MUS, treated mice (Group 2). CBDCA treatment (Group 3) resulted in 48.37% (P < 0.001) damaged cells with long comet tail formation. DNA with diffused head and scattered tail were also seen. MUS treatment during concomitant and pretreatment schedules significantly (P < 0.001) reduced the number of damaged cells by 66.09% and 74.36%, respectively in comparison with Group 3 mice. MUS administration also mitigated the comet tail length and Olive tail moment compared to the CBDCA-treated mice. In the case of 4 MU treatment group, the CBDCA-induced damage was lowered by 23.25% (P < 0.001) in bone marrow cell population.

In addition to the single cell gel electrophoresis, DNA fragmentation pattern of bone marrow cells in DNA gel electrophoresis (Fig. 2C) also confirmed the above findings. Single and clear band of genomic DNA was observed in the case of Group 1 (lane 2) and Group 2 (lane 3). And as expected, smear and ladder formation were seen in CBDCA-treated animals (Group 3, lane 4). This fragmentation pattern was markedly reduced in both the cases of concomitant and pretreatment schedule with MUS (lane 6 and 7 respectively). 4 MU pretreatment, however, was unable to prevent any DNA fragmentation (lane 5).

### Prevention of clastogenic catastrophe

When DNA damage reaches the threshold beyond the steady state, chromosomal aberrations are acquired over time^23^ which often leads to micronuclei formation. Biological consequences of DNA damage can be estimated by measuring CA and MN^24^. Animals treated with CBDCA showed significantly (P < 0.001) high micronuclei (MN) frequency (Fig. 2A1 and Table 1). Treatment with MUS in concomitant and pretreatment schedules was able to minimize the incidence of MN to a significant (P < 0.001) level by 67.63% and 76.30%, respectively. Administration of 4 MU significantly (P < 0.001) reduced the frequency of MN by 46.82%.

### Amelioration of reactive oxygen species and nitric oxide status and restoration of GSH and GSSG level

**Figure 3:**
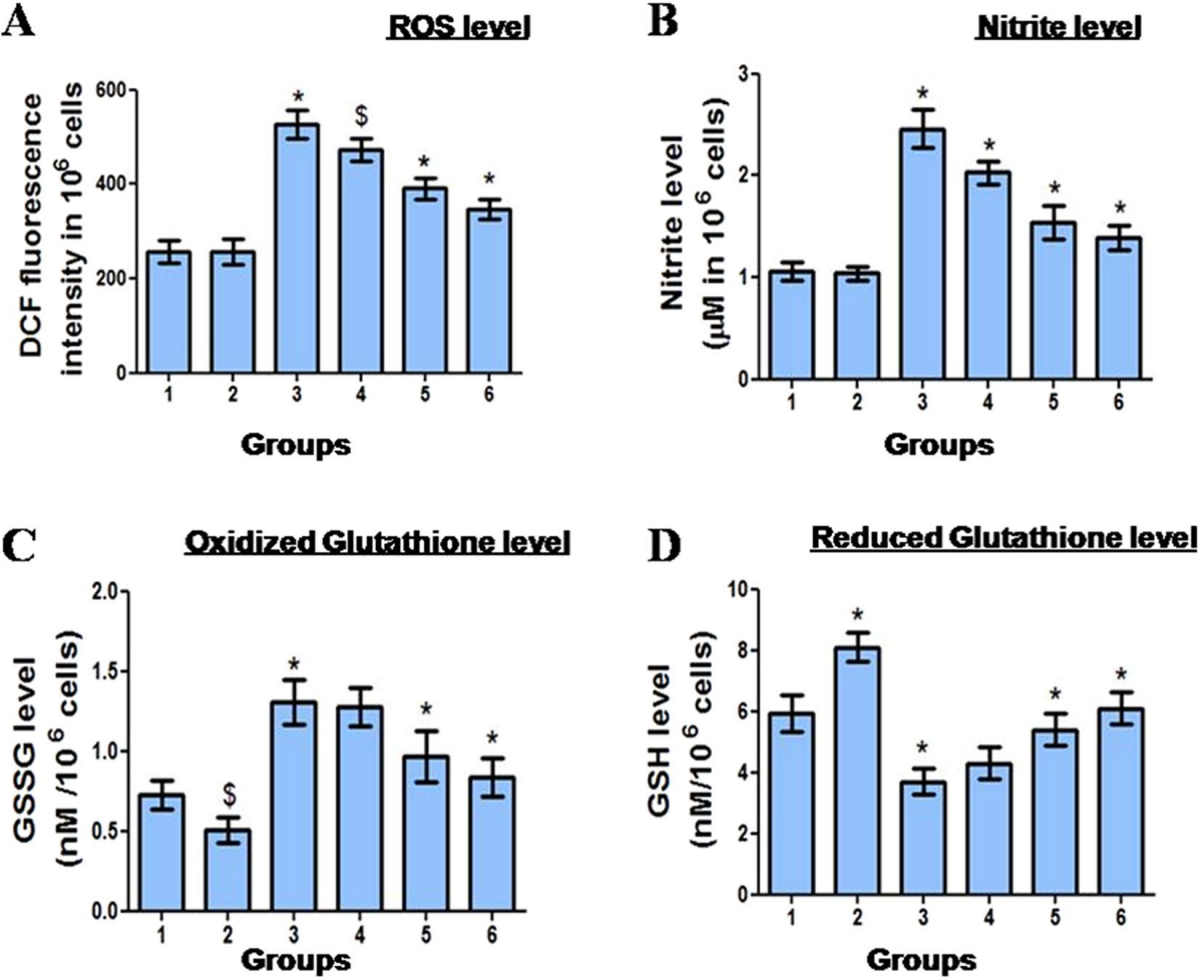
Modulation of CBDCA-induced redox imbalance by MUS in murine bone marrow cells. MUS reduced the levels of (**A**) ROS, **(B)** NO, **(C)** GSSG and enhanced the level of **(D)** GSH in bone marrow cells. Data are represented as mean ± SD, n = 6. *Denotes (P < 0.001) and ^$^denotes (P < 0.05) as compared to their respective control groups.

Administration of CBDCA resulted in a significant (P < 0.001) increase in the levels of ROS (Fig. 3A) and NO (Fig. 3B) in the bone marrow cells of the CBDCA control mice, in comparison to the vehicle-treated group, as measured by DCFH-DA and Griess reagent, respectively. In pretreatment scheme, MUS and 4MU administration reduced the levels of ROS by 34% (P < 0.001) and 9.93% (P < 0.05), respectively compared to the CBDCA-treated group. MUS in concomitant treatment significantly (P < 0.001) reduced the levels of ROS by 25.54%. MUS administration significantly (P < 0.001) prevented the CBDCA-induced rise in the level of NO by 37.39% and 43.49% in concomitant and pretreatment schedules respectively. Pretreatment with 4MU lowered the level of NO by 17.47% (P < 0.001). CBDCA administration significantly (P < 0.001) increased the level of GSSG by 79.45% (Fig. 3C) and decreased the level of GSH by 37.71% (P < 0.001) in comparison to vehicle-treated group (Fig. 3D). When applied in combination, MUS administration significantly (P < 0.001) reduced CBDCA-mediated increase of GSSG level by 25.95% and 35.87% in concomitant and pretreatment schedules, respectively. Treatment with 4 MU lowered (P > 0.05) the level of GSSG by 2.29% in comparison with CBDCA-treated animals. The level of GSH was significantly (P < 0.001) increased by 45.94% and 64.86% in MUS concomitant and pretreatment groups in comparison to CBDCA-treated group. Treatment with 4 MU increased (P > 0.05) the level of GSH by 16.21%.

### MUS effectively inhibited cancer cell proliferation *in vitro*

The growth inhibition in MCF-7 and Colo- 205 cell lines were analyzed by MTT assay to determine the *in vitro* therapeutic efficacy of CBDCA in combination with MUS (Table 2). Primarily the IC_50_ values of MUS in MCF- 7 and COLO- 205 were found to be 1.2 mM and 51.3 µM. Whereas the IC_50_ values of 4MU in MCF- 7 and COLO- 205 cell lines were 0.6 mM and 19.4 µM respectively. MUS and CBDCA each were combined at their corresponding IC_25_, IC_35_, IC_45_, IC_55_, IC_65_ and IC_75_ to get the Combination Index (CI) values from which mean CIs were estimated. The combination of 4MU and CBDCA was also tested in a similar way. We were delighted to found that, MUS and CBDCA interacted synergistically in both the cell lines with CIs being significantly lower than 1 (P < 0.05) (Table 2). Additionally, the interaction between 4 MU and CBDCA was also found synergistic in COLO-205 and additive in MCF- 7.

**Table 2:** Determination of IC_50_ values and Combination Index by MTT assay.

Cell lines	CBDCA	IC_50_ values		Combination index^*^	
		MUS	4 MU	CBDCA and MUS	CBDCA and 4 MU
MCF- 7	62.3 µM	1.2 mM	0.6 mM	0.72 ± 0.07	0.96 ± 0.08
COLO- 205	7.1 µM	51.3 µM	19.4 µM	0.38 ± 0.04	0.24 ± 0.04

Mean values calculated from combination indices achieved in the IC_25_–IC_75_ range of cytotoxicity.

### Cell death induced by MUS as measured by Annexin-V/FITC

**Figure 4:**
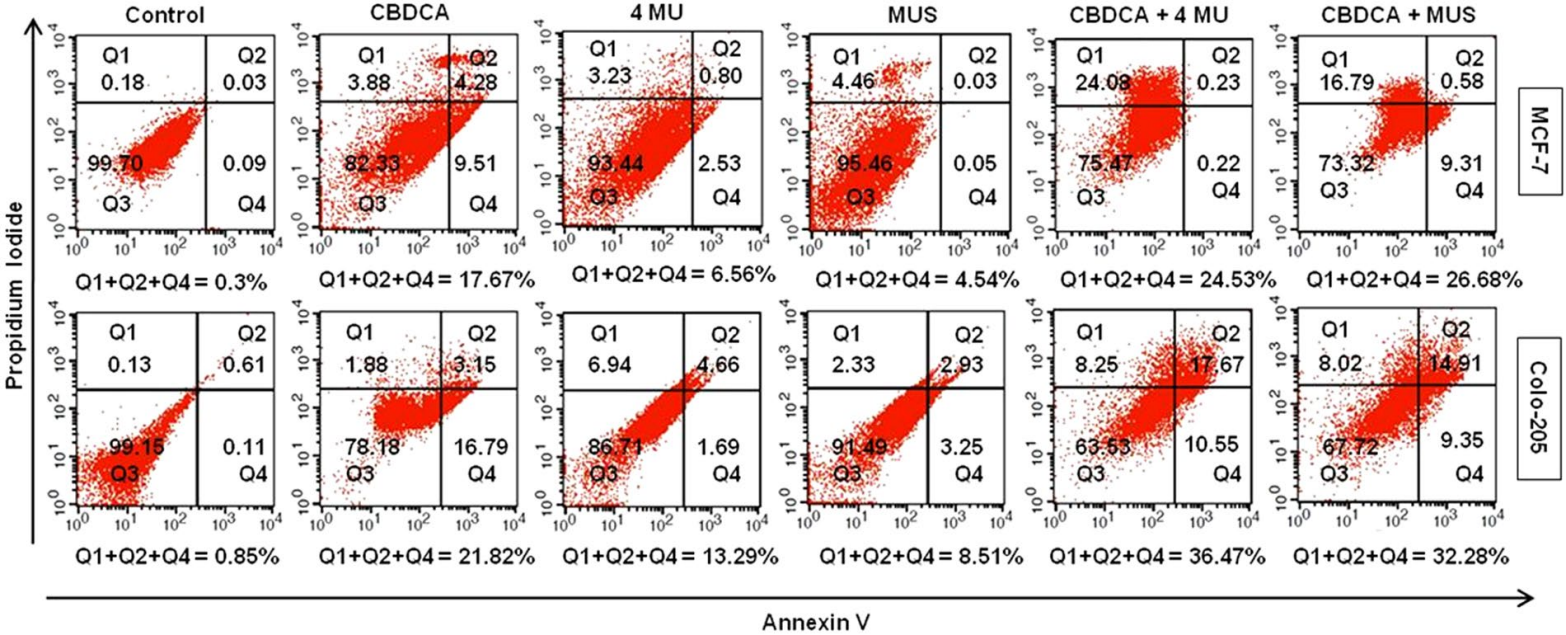
Effect of 4 MU and MUS in combination with CBDCA in MCF-7 and Colo-205 cell lines. After 48 h incubation with CBDCA, 4 MU, and MUS alone or in combination with their corresponding IC_25_, the cells were stained with annexin-V and PI as described in Methods and analyzed by flow cytometry. The figure is a representative profile of at least three experiments in duplicate.

In human cancer cell lines MCF-7 and Colo-205, 4MU showed high anti-proliferative potential than MUS. Cell death induced by CBDCA, MUS and 4 MU alone or in combination at their corresponding IC_25_ values were determined in MCF-7 and Colo-205 cell lines by annexin V/PI staining followed by flow cytometric analysis (Fig. 4). CBDCA, 4 MU and MUS, as a single agent, induced 17.67%, 6.56% and 4.54% cell death in MCF-7 cell line and 21.82%, 13.29% and 8.51% cell death in Colo-205 cell line respectively. Combination treatment of CBDCA and MUS induced 26.68% and 32.28% cell death in MCF-7 and Colo-205 cell lines. On the other hand, CBDCA and 4 MU-induced 24.53% and 36.47% cell death in these two cell lines. MUS synergistically interacted with CBDCA in both cell lines because calculated and observed cell death in MCF-7 were 22.21% and 26.68%, respectively, whereas in Colo-205 those were 30.33% and 32.28%, respectively. Interaction of 4 MU and CBDCA, however, was additive in MCF-7 cell line (calculated cell death- 24.23% and observed cell death-24.53%) and synergistic in the Colo-205 cell line (calculated cell death - 35.11% and observed cell death-36.47%).

### *In vivo* antitumour activity of MUS

**Figure 5:**
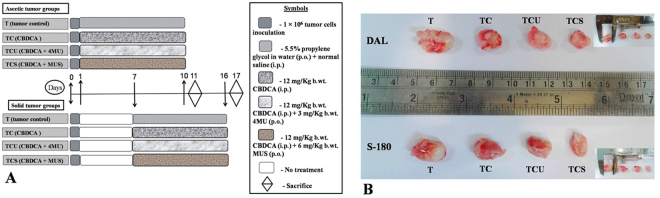
(**A**) Graphical presentation of different groups of mice for *in vivo* antitumour activity study. (**B**) Effect of 4MU and MUS in combination with CBDCA on tumour size of two different solid tumours (DAL and S-180). Measurement of tumour volume is shown in the inset.

In this present work, we have used two murine tumour cell lines *viz* Dalton’s Lymphoma (DAL) and Sarcoma-180 (S-180) to determine the *in vivo* antitumour potential of MUS in combination treatment. Moreover, ascites and solid tumour, both the forms were evaluated in the course of this study. The results revealed that MUS augmented the cytotoxicity of CBDCA in both the ascites tumour models of DAL and S-180. In the combination treatment group (TCS group) we observed reduction in the ascites tumour volume, packed cell volume as well as tumour cell count compared to only CBDCA treated animals (TC; Table 3). However, no significant changes in ascites tumour volume, packed cell volume and number of viable tumour cells were observed in 4-MU combination group (TCU group) (p > 0.05) as compared to only CBDCA treated animals. MUS in combination with CBDCA (TCS group) effectively reduced the DLA solid tumour (Table 4, Fig. 5) volume by 21.42% (P < 0.05) and DLA solid tumour weight by 23.53% (P < 0.01) as compared to TC group with tumour growth inhibitory response (TIR) of 38.09%. In case of S-180 solid tumour, however, we found statistically insignificant (p > 0.05) reduction of the tumour volume and weight in combination group (TCS) as compared to the CBDCA treatment as single agent. 4 MU in combination treatment could not significantly alter the solid tumour volume or tumour weight compared to the CBDCA, in any of the two solid tumour models studied.

**Table 3:** Effect of MUS on growth of two different ascites tumours in *vivo*.

Groups	Dalton’s Lymphoma					Sarcoma 180				
	Tumour volume (mL)	Packed cell vol. (mL)	Tumour cell count (×10^6^)	TIR (%)	Tumour cell inh. (%)	Tumour vol. (mL)	Packed cell vol (mL)	Tumour cell count (×10^6^)	TIR (%)	Tumour cell inh.(%)
T	5.3 ± 0.5	3.4 ± 0.4	47.1 ± 1.3	—	—	6.2 ± 0.4	4.1 ± 0.3	53.2 ± 1.2	—	—
TC	2.1 ± 0.3^*^	1.6 ± 0.2^*^	10.3 ± 1.1^*^	60.3	78.1	2.7 ± 0.2^*^	1.9 ± 0.1^*^	11.4 ± 1.0^*^	56.4	78.5
TCU	1.9 ± 0.2	1.3 ± 0.1	9.8 ± 0.7	64.1	79.1	2.3 ± 0.3	1.7 ± 0.2	10.9 ± 0.8	62.9	79.5
TCS	1.4 ± 0.1^#^	0.8 ± 0.1^*^	6.2 ± 0.5^*^	73.5	86.8	1.8 ± 0.2^*^	1.1 ± 0.1^*^	7.1 ± 0.6^*^	70.9	86.6

Data are represented as mean ± SD, n = 6. *Denotes (P < 0.001), ^#^denotes (P < 0.01) and ^$^denotes (P < 0.05) as compared to their respective control groups. Comparisons were made between T & TC group; whereas TCU and TCS were compared with TC group.

**Table 4:** Effect of MUS on growth of two different solid tumours in *vivo*.

Groups	Dalton’s Lymphoma			Sarcoma 180		
	Tumour Volume (cm^3^)	Tumour Weight (gm)	TIR (%)	Tumour Volume (cm^3^)	Tumour Weight (gm)	TIR (%)
T	1.8 ± 0.2	2.1 ± 0.3	—	1.9 ± 0.3	2.3 ± 0.2	—
TC	1.4 ± 0.1^#^	1.7 ± 0.1^#^	22.22	1.5 ± 0.1^#^	1.9 ± 0.1^$^	21.05
TCU	1.3 ± 0.2	1.6 ± 0.2	27.77	1.4 ± 0.2	1.8 ± 0.3	26.31
TCS	1.1 ± 0.1^$^	1.3 ± 0.1^#^	38.88	1.2 ± 0.1	1.6 ± 0.2	36.84

Data are represented as mean ± SD, n = 6. ^#^Denotes (P < 0.01) and ^$^denotes (P < 0.05) as compared to the respective control group. Comparisons were made between T and TC group; whereas TCU and TCS were compared with TC groups.

## Discussion

In the present investigation, we reported the synthesis and *in vivo* myeloprotective activity of a new organoselenium compound, MUS. Moreover, MUS was also found to potentiate the therapeutic efficacy of CBCDA *in vitro* and *in vivo*.

Acute oral toxicity threshold (LD_50_) of MUS (>2000 mg/Kg b.wt.) indicates the non-toxic nature^25^ of the compound. Dosage regimen was determined as per OECD guideline 407 as the best combination of administration route, dose amount and dose schedule can ensure the highest efficacy and lowest risk. As observed in this study, MUS at 6 mg/kg b.wt. p.o. helped to achieve overall good health, optimum antioxidative capacity without any clinically detectable toxicities. Hence this dose was identified as the most suitable dose to explore the protective capacity of MUS against CBDCA-induced myelotoxicity.

CBDCA induces apoptosis and reduces the proliferation of cancer cells. Due to lack of target specificity, it also damages bone marrow, a highly proliferative organ, resulting in myelosuppression. In this study, cell proliferation in bone marrow was notably reduced by CBDCA administration which was increased significantly after MUS treatment. Beside the proliferating segment, dying cell population in bone marrow was increased by CBDCA as measured by cell death and apoptotic index. MUS administration significantly reduced CBDCA induced cell death and apoptotic index in bone marrow cells. These results clearly demonstrated the cell survival promotion by MUS. Thus, the positive modulation of proliferation and survival resulted in higher viable bone marrow cell count.

Platinum is a heavy metal transition element and generally damages cellular DNA. In this present work, we have also observed such kind of DNA damage in bone marrow cells after treatment with CBDCA and MUS treatment able to reduce the laddering of DNA and comet formation. As shown, MUS was three times more effective in reducing the comet formation than 4 MU proves the role of selenium in preventing DNA damage. Selenium in various forms (i) is reported to enhance biochemical response against DNA damage *in vitro, in vivo* and in clinical trials;^26^ (ii) can prevent the induction of DNA damage and (iii) increase the activity of DNA repair enzymes^27^. Although not conclusive, but in most of the cases DNA repairing system was strengthened rather than increased removal of DNA adduct to cope up with these assaults. It is also documented that, the incidence of DNA damage from oxidative injury are reduced due to the activity of glutathione peroxidase, a well-known selenoprotein that maintains redox balance^28^. In the present study, the severity of CBDCA induced DNA damage was observed to be reduced after MUS administration. We also observed that reduction in DNA damage led to the lowering of chromosomal aberrations and subsequent micronuclei formation in bone marrow cells.

We assumed that myelotoxicity of CBDCA may be caused by the free radicals mediated assault to DNA in murine bone marrow cells because (i) CBDCA induced free radicals are reported to be responsible for neurotoxicity, ototoxicity^29^ and nephrotoxicity^3^, (ii) heavy metals are known to generate free radicals which can interact with DNA and produce lesions^30^ and (iii) CBDCA can intercalate within DNA that resulted in the formation of the free radicals generated by platinum can damage DNA in spite of their short lifespan. We examined this hypothesis by measuring cellular oxidative and nitrosative stress. As expected, the oxidative burst was observed in CBDCA-treated murine bone marrow cells (Fig. 3A,B). The increment in the levels of cellular ROS and NO level in CBDCA-treated bone marrow cells was also accompanied by an increase in the level of GSSG and decrease in the level of cellular GSH (Fig. 3C,D). Administration of MUS lowered the free radicals generation, lowered the level of GSSG, elevated the cellular level of GSH and thereby recovered murine bone marrow cells from oxidative and nitrosative injury. The antioxidative potential of MUS which was observed during dose selection (Suppl Table 3), can easily explain these findings. So the cytoprotection conferred by MUS may be attributed to the selenium moiety in MUS.

MUS alone were found to be less cytotoxic towards transformed cells than 4 MU *in vitro*. But MUS in combination with CBDCA resulted in better synergistic cytotoxicity *in vitro*. This may be attributed to the fact that MUS regulates other antiproliferative pathways due to the presence of selenium moieties. *In vivo* studies revealed that administration of MUS in combination with CBDCA significantly increased the cytotoxicity of CBDCA against two murine cancer cell lines i.e. DLA and S-180. As the anti-cancer activity of CBDCA is ROS-dependent, so MUS must behave like a prooxidant wherein oxidative environment of cancer cells acted as catalyst resulting in synergistic cytotoxicity. But in a homeostatic environment of normal bone marrow cells, MUS was found to be antioxidant while interacted with CBDCA. This dual behaviour of MUS may be due to the presence of selenium moiety in MUS since selenium compounds are well known to act as antioxidants and pro-oxidants depending upon cellular redox state^31^. Tumour cells generally possess a high level of ROS. Selenium compounds due to its characteristic pro-oxidant property in tumour microenvironment can act as selective cytotoxic agents against cancer cells by achieving toxic levels of ROS. However, the chemical form of selenium and the dose employed often dictate the interplay between the pro-oxidant and antioxidant behaviour of selenium compounds. Higher concentration of selenium was proved to act as pro-oxidant due to the generation of ROS^32,33^. On the other hand, the higher bioavailability of organic selenium than inorganic selenium made the former one a potential candidate for preclinical and clinical development. Moreover, the metabolism of different forms of selenium also plays a key factor in delivering the pro-oxidant and antioxidant efficacy of selenium^34–36^.

In summary, we have synthesized a new hit, MUS which showed myeloprotective efficacy in combination with CBDCA and also synergizes the therapeutic efficacy of the standard chemotherapeutic drug. The underlying molecular mechanism of MUS is underway for further modification of its structure to produce the potential lead molecule.

## Methods

### Materials

MCF -7 and Colo-205 cell lines were brought from NCCS, Pune, India. CBDCA was purchased from Fresenius Kabi India Pvt. Ltd., Pune, India. All reagents were purchased from commercial suppliers and used directly unless otherwise stated.

### Synthesis and characterization of MUS

**Figure 6:**
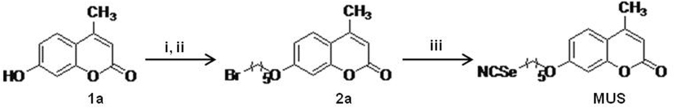
Synthesis of MUS-reagents and conditions. (i) anhydrous potassium carbonate, dry acetone, refluxed for 2 h under N_2_; (ii) 1,5-dibromopentane, reflux 9 h (iii) KSeCN, 48 h, rt.

The Methyl-substituted umbelliferone selenocyanate (MUS) was synthesized following a two-step procedure as shown in Fig. 6. A mixture of 7-hydroxy-4-methyl-2H-chromen-2-one (1a) (1.5 g, 9.2 mM) and anhydrous potassium carbonate (2.5 g, 18.4 mM) in dry acetone was refluxed for 2 h under N_2_ atmosphere. After cooling to room temperature, 1,5- dibromopentane (4.2 ml, 18.4 mM) was added to the mixture and refluxed for another 9 h. The resulting bromo compound (**2a**) was extracted with ethyl acetate and purified by column chromatography using petroleum ether/ethyl acetate. The desired selenocyanate (MUS) was then obtained by dropwise addition of a solution of anhydrous potassium selenocyanate (0.64 g, 4.5 mM) in acetone to a stirred suspension of the precursor bromo-compound (1.17 g, 3.6 mM) over a period of 1 h at 25 °C. The reaction mixture was stirred for 48 h at room temperature (reaction was monitored by TLC).The product (**MUS**) was extracted with CHCl_3_ and purified by column chromatography using petroleum ether/chloroform.

Compound **2a:** Yield: 57%. m.p: 63.1 °C.

^1^H NMR (600 MHz, CDCl3) δ 7.45 (1 H, d, J = 8.64 Hz), 6.85 (1 H, d, J = 8.4 Hz), 6.74 (1 H, s), 6.09 (1 H, s), 3.99 (2 H, t, J = 5.8 Hz), 3.40 (2 H, t, J = 6.4 Hz), 2.36 (3 H, s), 1.92 (2 H, q, J = 6.9 Hz), 1.82 (2 H, q, J = 6.9 Hz) and 1.64–1.60 (2 H, m). (suupl Fig. 1)

^13^C NMR (150 MHz, CDCl_3_) δ 162.08, 161.31, 155.29, 152.55, 125.52, 113.54, 112.61, 111.92, 101.37, 68.18, 33.45, 32.39, 28.19, 24.75 and 18.66. (suupl Fig. 2)

MS (ESI+) m/z calcd C_15_H_18_BrO_3_+ [M + H]+ , 325.04 and 327.04; found 325.38 and 327.38.

Compound **MUS**: Yield: 62%; m.p.71 ^o^C.

IR (KBr) υ_max_ cm^−1^: 2943.31, 2860.96, 2147.24, 1708.57, 1692.7, 1608.33.

^1^H NMR (800 MHz, CDCl_3_) δ 7.48 (d, J = 8.8 Hz, 1 H), 6.88–6.80 (m, 1 H), 6.80–6.73 (m, 1 H), 6.11 (s, 1 H), 4.02 (t, J = 6.2 Hz, 2 H), 3.08 (t, J = 7.5 Hz, 2 H), 2.38 (s, 3 H), 1.99 (t, J = 7.6 Hz, 2 H), 1.86 (t, J = 7.5 Hz, 2 H), 1.65 (d, J = 7.8 Hz, 2 H). (suupl Fig. 3)

^13^C NMR (201 MHz, CDCl_3_) δ 161.93, 161.34, 155.22, 152.66, 125.60, 113.58, 112.55, 111.90, 101.51, 101.35, 67.99, 30.62, 29.26, 28.29, 25.73, 18.71.(suupl Fig. 4)

HRMS (ESI+) m/z calcd C_16_H_18_NO_3_Se+ [M + H]+ , 352.0452; found 352.3062; C_16_H_17_NNaO_3_Se+ [M + Na]+ , 374.0271; found 374.3431.

### Experimental animals

Adult (6–8 weeks) Swiss albino female mice (25 ± 2 g) used in this study were bred and maintained in the animal colony of Chittaranjan National Cancer Institute (Kolkata, India). The mice were maintained at a controlled temperature (22 ± 2 °C) and humidity (60 ± 5%) under alternating light and dark conditions (12 h/12 h) in standard cages. Standard food pellets and drinking water were provided *ad libitum*. Animals were acclimatized for 7 days before all the starting of an experiment. Experiments like blood sample collection and sacrifice of the animals were performed by strictly following the standard guidelines of Institutional Animal Ethics Committee [Committee for the Purpose of Control and Supervision of Experiment on Animals (CPCSEA Registration No. 1774/GO/RBi/S/14/CPCSEA), India]. All the experimental methods and protocols were approved and in accordance with the Institutional Human Ethics Committee, Chittaranjan National Cancer Institute, Kolkata.

### Median lethal dose (LD_50_) determination

Oral LD_50_ of MUS was determined by Up-and-Down-Procedure as per the OECD guidelines 425. After administration of each dose, animals were continuously observed for the first 4 h and then periodically up to 48 h for any adverse symptoms. The animals were kept under observation for next 14 days for any delayed mortality. The oral LD_50_ of MUS at 95% confidence interval was analyzed by AOT425 software according to OECD guideline.

### Dose selection of MUS

MUS was orally administered as a suspension of 5.5% propylene glycol in water. Animals were divided into four groups, containing six animals (n = 6) in each group. Vehicle control group: Each animal was given 5.5% propylene glycol in water for 28 days. MUS (3 mg/kg b.wt.)- treated group: The compound was orally administered at the dose of 3 mg/kg b.wt. for 28 days. MUS (6 mg/kg b.wt.)- treated group: The compound was orally administered at the dose of 6 mg/kg b.wt. for 28 days. MUS (12 mg/kg b.wt.)- treated group: The compound was orally administered at the dose of 12 mg/kg b.wt. for 28 days. The animals were treated for 28 days and sacrificed on the 29^th^ day, 24 h after last treatment.

### Experimental groups for chemoprotection study

The animals were divided into six groups (Fig. 1A), containing six animals (n = 6) in each group. *Group 1* (vehicle-treated group): 5.5% propylene glycol in water was administered orally for 17 days along with the intraperitoneal injection of normal saline from day 8 to 17. *Group 2* (MUS only treated group): MUS in 5.5% propylene glycol was orally administered at 6 mg/kg b.wt. for 17 days. *Group 3* (CBDCA treated group): CBDCA was intraperitoneally administered at 12 mg/kg b.wt. from day 8 to 17. *Group 4* (4 MU pretreatment group): 4MU in 5.5% propylene glycol was orally administered at 3 mg/kg b.wt. for 17 days along with the intraperitoneal injection of CBDCA at 12 mg/kg b.wt. from day 8 to 17. *Group 5* (MUS concomitant treatment group): MUS in 5.5% propylene glycol was orally administered at 6 mg/kg b.wt. from day 8 to 17 along with the intraperitoneal injection of CBDCA at 12 mg/kg b.wt. from day 8 to 17. *Group 6* (MUS pretreatment group): MUS in 5.5% propylene glycol was orally administered at 6 mg/kg b.w. for 17 days along with the intraperitoneal injection of CBDCA at 12 mg/kg b.wt. from day 8 to 17. All experimental animals were sacrificed on the 18^th^ day, 24 h after last treatment.

### Sample collection

Before sacrifice, all animals were fasted for 4 h and anaesthetized with i.p. injection of ketamine-xylazine solution (87.5 mg/kg b.wt. ketamine and 12.5 mg/kg b.wt. xylazine). Blood samples from retro-orbital venous plexus were collected in a heparinized vial and subjected to haematological analysis. Cells from spleen, thymus and femoral bone marrow were collected by aspirating with 1X PBS through 26 gauge needle.

### *In situ* cell proliferation detection by BrdU Labeling

1 × 10^6^ cells/ml were incubated with BrdU in RPMI at 37 °C in a humidified incubator with 5% CO_2_ for 1 h. Then the cells were smeared and fixed on slides followed by incubation with anti-BrdU monoclonal antibody at 37 °C for 30 min. Alkaline phosphatase conjugated anti-mouse immunoglobulin was then added to it followed by 30 min incubation at 37 °C. The slides were then incubated with BCIP/NBT substrate solution at 25 °C for 30 min and visualized under a light microscope^37^. Cells (≥100) were counted from the randomly selected area of each slide and percentages of labelled cells were expressed as BrdU labelling index.

### Chromosomal aberration

0.03% colchicine (10 mL/kg b.w., i.p.) was administered 90 min before sacrifice. Bone marrow cells were collected in warmed (37 °C) 1% sodium citrate solution and fixed in acetic acid/ methanol (1:3) on slides. After flame drying and Giemsa staining^38^, slides were scored under a light microscope at ×1000 magnification. Percentage of aberrated metaphase plate was noted as a chromosomal aberration (CA).

### Micronuclei (MN) detection

The cells from the femoral bone marrow were flushed out by 0.075 M KCl solution and incubated at 37 °C for 10 min. After which the cells were harvested by centrifugation, smeared on slides, air-dried and fixed in absolute methanol. Following Giemsa staining^38^, the slides were visualized under a light microscope at ×1000 magnification. Nuclei with a diameter between 1/16 and 1/3 of the original nucleus which was on the same plane, stained uniformly and non-overlapping with an original nucleus are considered as micronuclei. The percentage of MN in each group was reported after a counting of ≥1000 cells from randomly selected zones on the slide.

### Comet assay

The femoral bone marrow cells (2 × 10^4^) was layered in 1.0% low melting agarose onto half-frosted slides pre-coated with a thin layer of 1.0% normal melting agarose^39^. The third layer of 0.5% low melting agarose was added to the top. The slides were kept for 2 h at 4 °C in lysis solution (2.5 M NaCl, 100 mM EDTA, 10 mM Tris, 10% dimethyl sulfoxide, 1% Triton X-100,pH 10.0) after which electrophoresis was performed for 30 min in buffer (1 mM EDTA, 0.3 M NaOH, pH 13.1). Then the slides were neutralized (0.4 M Tris-HCl, pH 7.5), dried at room temperature and stained with ethidium bromide. Various parameters^40^ like damaged cell (%), average tail length [migration of the DNA from the nucleus (μm)] and Olive tail moment [product of tail length and the fraction of total DNA in the tail (arbitrary units)] were measured using KOMET 5.5 software, Andor Technology, USA.

### Genomic DNA integrity testing by Agarose gel electrophoresis

Genomic DNA from femoral bone marrow cells (1 × 10^6^) was isolated using HiPurA^TM^ Multi-sample DNA Purification Kit as per manufacturer instruction. Electrophoresis was performed in 1.5% agarose gel containing ethidium bromide (0.5 μg/ml) along with 1 kb DNA ladder. Photograph of banding patterns was taken by a gel documentation system.

### *In situ* cell death estimation by TUNEL assay

Femoral bone marrow cells were smeared on slides, permeabilized using 0.1% Triton X-100 and then incubated with TUNEL reaction mixture containing the TdT (Terminal Deoxynucleotidyl Transferase) and fluorescein-dUTP (2′-deoxyuridine 5′-triphosphate) at 37 °C for 1 h in a humidified chamber^41^. The slides were then analyzed in a fluorescence microscope and photomicrographs were taken at ×400 magnifications. The apoptotic cells were identified by green fluorescence. Randomly selected cells (≥100) from 5–6 zones/slide were counted to determine the number of apoptotic cells. Apoptotic index (AI) was determined as the percentage of the labelled nuclei with respect to the total number of nuclei counted.

### Quantification of hydroxyl and nitric oxide level

10 μM DCFH-DA was added in femoral bone marrow cells (2 × 10^6^) and incubated in dark for 30 min at 25 °C to allow the formation of DCF^42^ which was analyzed (excitation at 485 nm, emission at 529 nm) using spectrofluorometer to measure the levels of ROS. The level of nitrate (NO_3_^−^) and nitrite (NO_2_^−^) in 1 × 10^6^ bone marrow cells were measured by adding Griess reagent (1% sulphanilamide, 5% phosphoric acid and 0.1% NEDD) to it^43^. Absorbance at 545 nm was measured using NaNO_2_ as standard.

### Measurement of the levels of GSH and GSSG

The levels of GSH and GSSG in femoral bone marrow cells (2 × 10^6^) were assessed by a kinetic assay^44^ in which the catalytic amounts of GSH caused a continuous reduction of DTNB to TNB at 412 nm. Quantification was done by parallel estimation of standard curves of known GSH and GSSG concentrations.

### *In vitro* cell viability assay and analysis of the interactions

The Anti-proliferative potential of 4MU, MUS, and CBDCA alone or in combination was determined by MTT assay. MCF-7 and Colo-205 cells were seeded (1 × 10^4^ cells) into 96-well plates and incubated at 37 °C in a humidified incubator with 5% CO_2_ for 24 h. Different concentrations of 4MU, MUS, and CBDCA in media were added to each well and incubated for 48 h. Then MTT (0.4 mg/ml) solution was added to each well followed by 4 h incubation. After removal of the supernatant, the Formosan crystals formed in the cell were dissolved in DMSO and the absorbance was measured by multiwell spectrophotometer at 570 nm. Cell viabilities were expressed as percentages relative to untreated controls.

MUS and CBDCA each were added at their corresponding IC_25_, IC_35_, IC_45_, IC_55_, IC_65_ and IC_75_ in combination to get the Combination Index (CI) values^45^ from which mean CIs were estimated. The effect of combined cytotoxicity was categorized by combination index method (CIs) using the following equation: CI = (D_MUS_/D_MUS_x) + (D_CP_ /D_CP_x) + (D_MUS_ × D_CP_/D_MUS_x × D_CP_x), with D_MUS_, D_CP_ being each drug (MUS, carboplatin) concentration in the mixture, required to induce x% cytotoxicity and D_MUS_x, D_CP_x is the concentration of each drug required to induce the same cytotoxicity when used alone. The interaction between 4MU and MUS is considered synergistic if CI < 1, additive if CI = 1 and antagonistic if CI > 1.

### Cell death estimation by annexin V/PI staining

5 × 10^4^ cells (MCF-7 and Colo-205) were seeded in 6-well plates. After 80% confluent growth, CBDCA, MUS and 4 MU were added in their corresponding IC_25_ values alone and in combinations, and incubated for 48 h, after which the cells were harvested. In the case of *in vivo* experiment, femoral bone marrow cells were isolated and harvested by centrifugation. Thereafter apoptotic or necrotic cell death (*in vitro* and *in vivo*) was determined by using Annexin-V/PI kit (BD Biosciences, Kolkata). Briefly, 1 × 10^6^ cells were suspended in 100 μl of binding buffer. After that Annexin V-FITC and propidium iodide (PI) was added in 1:1 ratio and incubated at room temperature for 15 min in dark. 400 μl of binding buffer was then added and percentages of apoptotic or necrotic cells were analyzed with a flow cytometer.

### *In vivo* antitumour activity

Anti-tumour activity was evaluated *in vivo* (ascites and solid tumour) against two murine cancer cell lines *viz* Dalton’s Lymphoma (DAL) and Sarcoma-180 (S-180). These cell lines were maintained in Swiss albino mouse by intraperitoneal transplantation of 1 × 10^6^ viable tumour cells once in a week. In each cancer model (two ascites and two solid), animals were divided into four groups (Fig. 5), containing six animals (n = 6) in each. 1 × 10^6^ tumour cells/mouse were used to generate a tumour (i.p. injection for the ascites model and subcutaneous injection into left flank for solid tumour). The day of tumour cell inoculation was counted as “day 0”. In the ascites tumour model, treatment was started from day 1 and in solid tumour model treatment was started after 7 days (i.e palpable tumour formation). The experimental groups were divided as below-

T (tumour control): 5.5% propylene glycol in water (p.o.) and normal saline (i.p.) for 10 days.

TC (CBDCA treated group): 12 mg/Kg b.wt. CBDCA (i.p.) for 10 days.

TCU (CBDCA + 4MU treated group): 4MU was given ate dose of 3 mg/kg b.wt. in 5.5% propylene glycol (p.o.) and CBDCA at the dose of 12 mg/Kg b.wt. (i.p.) for 10 days.

TCS (CBDCA + MUS treated group): MUS was given ate dose of 6 mg/kg b.wt. in 5.5% propylene glycol (p.o.) and CBDCA at the dose of 12 mg/Kg b.wt. (i.p.) for 10 days.

All experimental animals were sacrificed 24 h after the last treatment (11^th^ day for the ascites tumour and 17^th^ day for the solid tumour). After sacrifice, ascitic tumour cells and solid tumour tissues were collected and washed in isotonic PBS solution.

In ascites models, the percentage of tumour cell inhibition (%) was calculated as (A − B)/A × 100, where ‘A’ represents the mean tumour cell count of the respective control group and ‘B’ represents the mean tumour cell count of the treated group. The solid tumour volume was calculated as tumour volume (cm^3^) = 4/3πr_1_^2^ × r_2_; where r_1_ is the width and r_2_ is the length of the solid tumour measured by Vernier calliper. Tumour-growth inhibition rate (% TIR) was calculated according to the formula (P − Q)/P × 100, where ‘P’ represents the mean tumour volume of the respective control group and ‘Q’ represents the mean tumour volume of the treated group.

### Statistical analysis

All data were expressed as mean ± SD, n = 6 mice per group. The mean values were statistically analyzed by One-way Analysis of Variance using SPSS 11 followed by Dunnett’s multiple comparison post hoc tests. A significant difference was indicated when the p-value was <0.05.

### Data availability

The data obtained in anonymized subjects, the full dataset, statistical details are available from the corresponding author (SB).

## Electronic supplementary material

Supplementary Information
