# Supplementary material for: Design and synthesis of coumarin-based organoselenium as a new hit for myeloprotection and synergistic therapeutic efficacy in adjuvant therapy
Source: Sci Rep. 2018 Feb 1;8:2194. doi: 10.1038/s41598-018-19854-5 (PMC5794964; doi:10.1038/s41598-018-19854-5)
Supplement: Supplementary file 1 — Supplementary Information [file 41598_2018_19854_MOESM1_ESM.doc]

**Design and synthesis of coumarin-based organoselenium as a new hit for myeloprotection and synergistic therapeutic efficacy in adjuvant therapy**

Arup Ranjan Patra^a^, Somnath Singha Roy^b^, Abhishek Basu^a^, Avishek Bhuniya^c^, Arin Bhattacharjee^a^, Subhadip Hajra^a^, Ugir Hossain Sk.^d^, Rathindranath Baral^c^, Sudin Bhattacharya^a*^

^a^Department of Cancer Chemoprevention, Chittaranjan National Cancer Institute, 37, S. P. Mukherjee Road, Kolkata- 700 026, West Bengal, India.

^b^Centre of Biomedical Research, Sanjay Gandhi Post-Graduate Institute of Medical Sciences Campus, Raebareli Road, Lucknow, 226014, India.

^c^Department of Immunoregulation and Immunodiagnostics, Chittaranjan National Cancer Institute, 37, S. P. Mukherjee Road, Kolkata- 700 026, West Bengal, India.

^d^Natural Product Chemistry & Process Development Division, CSIR-Institute of Himalayan Bioresource Technology, Palampur-176061, Himachal Pradesh, India.

*Correspondence:

Dr. Sudin Bhattacharya, Department of Cancer Chemoprevention, Chittaranjan National Cancer Institute, 37, S.P. Mukherjee Road, Kolkata–700 026, West Bengal, India. Tel: +91-33-24765101 (extn. 316). E-mail: sudinb19572004@yahoo.co.in


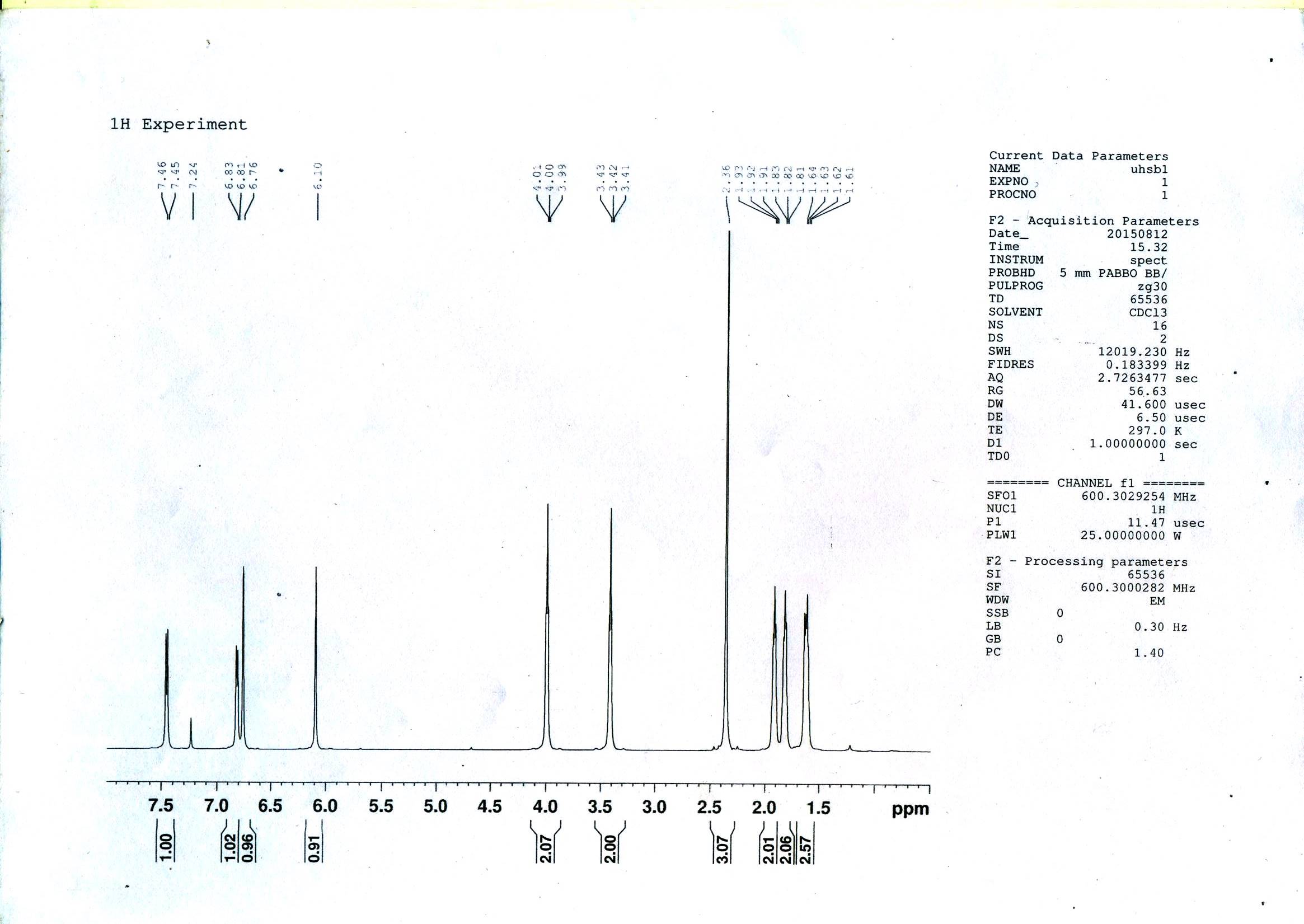
**Supplementary information**


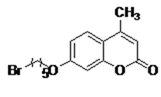


**Suppl Fig 1. 1H NMR data of 2a**


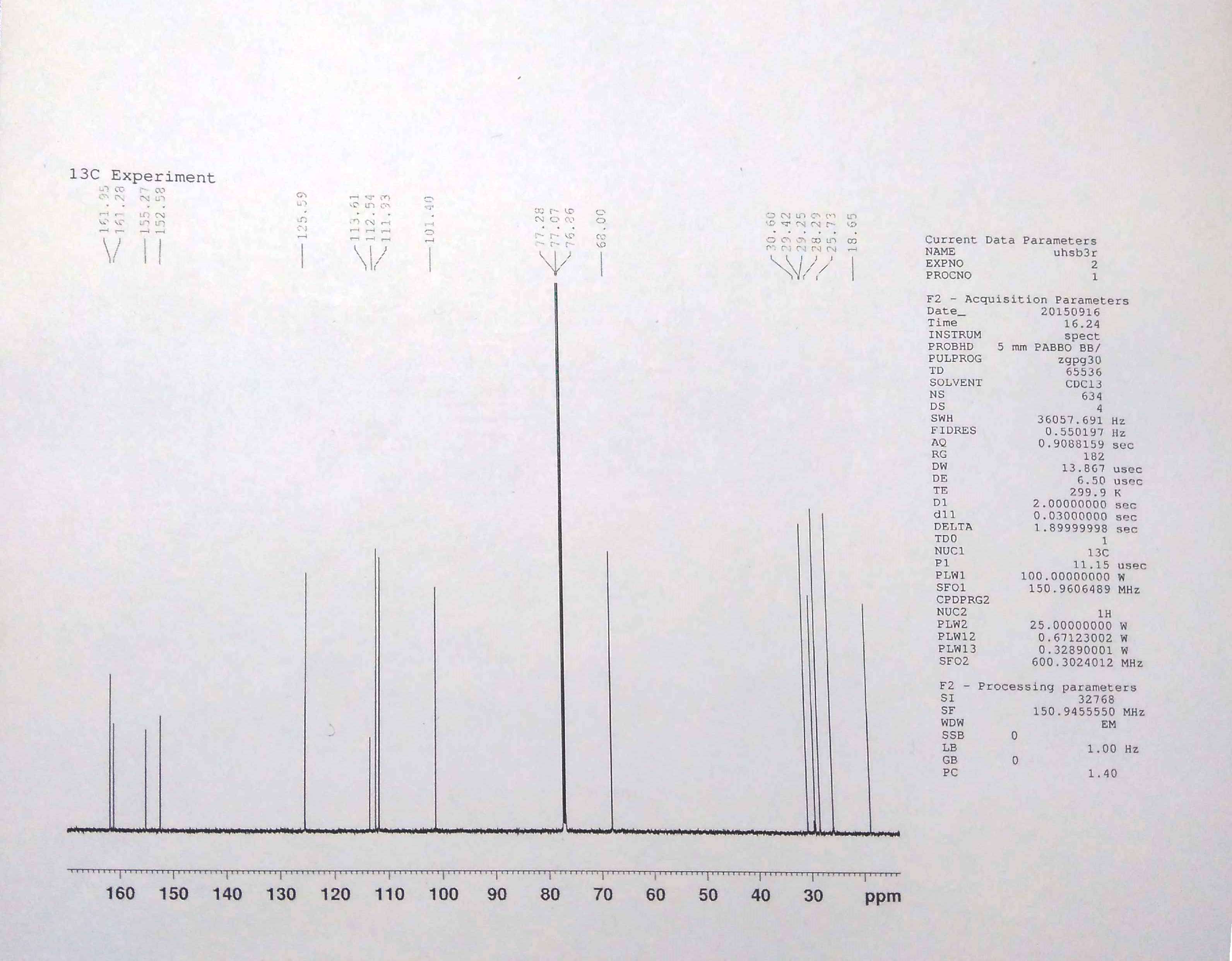


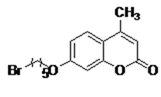


**Suppl Fig 2. 13C NMR data of 2a**

**
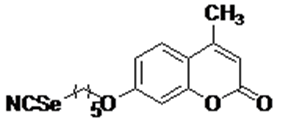
**

**Suppl Fig 3. 1H NMR data of MUS**

**
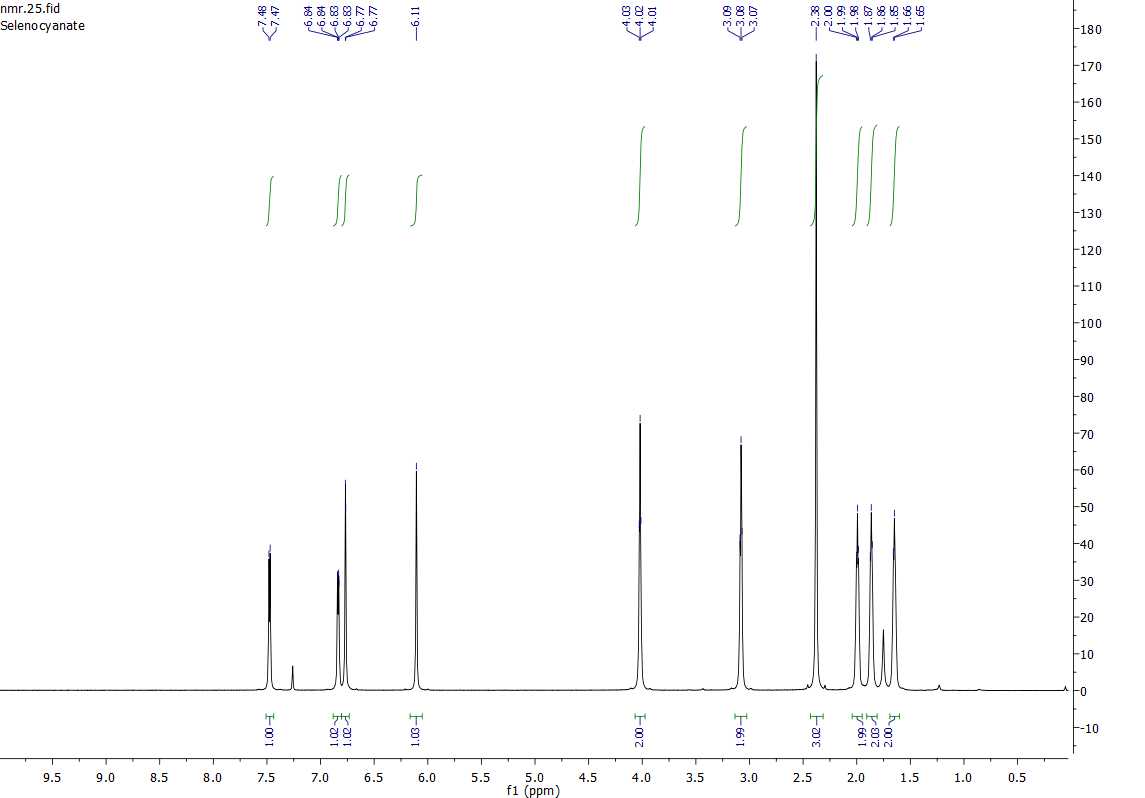
**

**Suppl Fig 4. 13C NMR data of MUS**
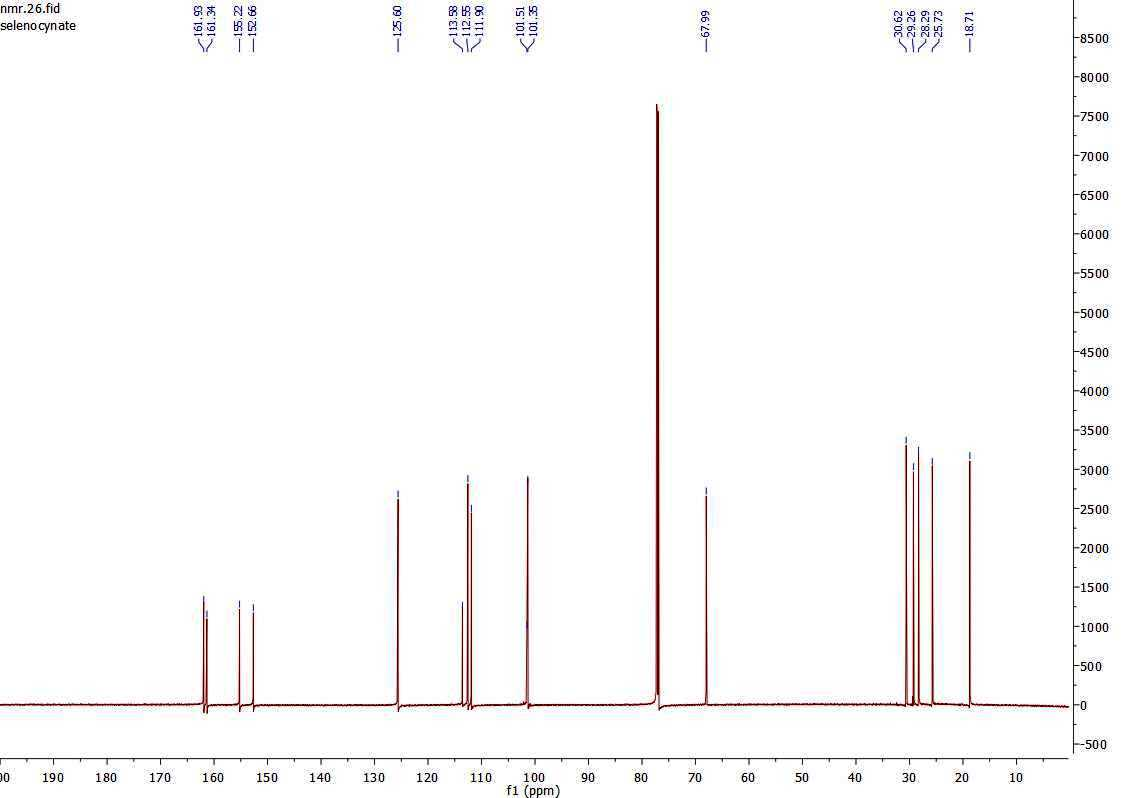

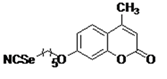


**Suppl Table 1**: Effect of MUS at different doses on body weight, hepatic LPO level, serum ALT, AST activity, BUN, creatinine level and CK, CK-MB activity following 28 days exposure.

| **Groups** | **Body weight (gm)** | **Hepatic** **LPO**  **(nmTBARS/mg protein)** | **ALT (IU/ml)** | **AST (IU/ml)** | **BUN (mg/dl)** | **Creatinine (mg/dl)** | **CK (U/L)** | **CK-MB (U/L)** |
| --- | --- | --- | --- | --- | --- | --- | --- | --- |
| Vehicle-control group | 25.81 ± 1.37 | 0.27 ± 0.02 | 41.27 ± 2.91 | 114.38 ± 11.62 | 19.17 ± 1.35 | 0.74 ± 0.06 | 137.66± 11.29 | 25.76± 2.17 |
| MUS (3 mg/kg b.wt.)-treated group | 25.94 ± 1.63 | 0.26± 0.01 | 40.95 ± 3.71 | 112.87 ± 7.43 | 18.44 ± 0.97 | 0.72 ± 0.09 | 131.65± 11.37 | 24.73± 2.82 |
| MUS (6 mg/kg b.wt.)-treated group | 26.37± 1.29 | 0.25± 0.03 | 40.03 ± 2.43 | 110.92 ± 9.41 | 18.23 ± 1.41 | 0.71 ± 0.08 | 129.43± 10.61 | 24.21± 2.94 |
| MUS (12 mg/kg b.wt.)-treated group | 25.63± 1.41 | 0.32 ± 0.04^#^ | 48.53 ± 2.16^*^ | 132.93 ± 8.71^*^ | 27.28 ± 1.63^*^ | 0.97 ± 0.11^*^ | 190.52± 16.43^*^ | 31.19± 1.71^#^ |

Data are represented as mean ± SD, n = 6.* denotes (P < 0.001) and # denotes (P < 0.01) as compared to vehicle-treated group.

**Suppl Table 2**: Effect of MUS at different doses on hematological parameters and cell count in different organs following 28 days treatment.

| **Groups** | **Hb (gm/dL)** | **RBC (10^6^/mm^3^)** | **WBC**  **(10^3^/mm^3^)** | **Neutrophil (%)** | **Lymphocyte (%)** | **Bone Marrow cell Count × 10^6^ /femur** | **Spleen cell Count × 10^6^** | **Thymus cell Count × 10^6^** |
| --- | --- | --- | --- | --- | --- | --- | --- | --- |
| Vehicle-control group | 13.63 ± 0.54 | 8.11 ± 0.32 | 7.62 ± 0.59 | 12.1 ± 0.68 | 73.2 ± 8.21 | 9.85 ± 1.11 | 15.58 ± 1.24 | 9.45 ± 1.01 |
| MUS (3 mg/kg b.w.)-treated group | 13.70 ± 0.36 | 8.19 ± 0.27 | 7.71 ± 0.43 | 12.3 ± 0.71 | 75.1 ± 7.19 | 10.60 ± 1.13 | 16.71 ± 1.63 | 10.28 ± 1.11 |
| MUS (6 mg/kg b.w.)-treated group | 14.05 ± 0.29 | 8.71 ± 0.34^$^ | 7.83 ± 0.31 | 12.7 ± 0.53 | 76.3 ± 5.87 | 11.38 ± 1.21 | 17.52 ± 1.53 | 11.20 ± 0.97 |
| MUS (12 mg/kg b.w.)-treated group | 12.97 ± 0.43 | 7.76 ± 0.35 | 7.85 ± 0.62 | 13.2 ± 0.57 | 78.9 ± 6.42 | 11.45 ± 1.60 | 18.52 ± 1.24^$^ | 14.00 ± 1.32^$^ |

Data are represented as mean ± SD, n = 6. $ denotes (P < 0.05) as compared to vehicle-treated group.

**Suppl Table 3**: Effect of MUS at different doses on hepatic GSH level and SOD, CAT, GST and GPx activity following 28 days exposure.

| **Groups** | **GSH (nM/mg protein)** | **SOD (U/mg protein)** | **Catalase (U/mg protein)** | **GST****(nM CDNB-GSH conjugate formed/min/mg protein)** | **GPx (µM NADPH utilized/min/mg protein)** |
| --- | --- | --- | --- | --- | --- |
| Vehicle-control group | 64.38 ± 7.23 | 219.43 ± 18.27 | 33.79 ± 4.38 | 389.54 ± 21.42 | 0.47 ± 0.06 |
| MUS (3 mg/kg b.wt.)-treated group | 72.86 ± 6.31 | 237.91 ± 23.14 | 34.92 ± 3.21 | 392.28 ± 19.63 | 0.51 ± 0.08 |
| MUS (6 mg/kg b.wt.)-treated group | 91.49 ± 8.56^*^ | 261.24 ± 19.63^$^ | 47.62 ± 5.43^*^ | 486.55 ± 23.19^*^ | 0.68 ± 0.07^*^ |
| MUS (12 mg/kg b.wt.)-treated group | 63.41 ± 9.72 | 223.82 ± 21.39 | 32.53 ± 3.64 | 386.93 ± 18.97 | 0.43 ± 0.05 |

Data are represented as mean ± SD, n = 6.* denotes (P < 0.001) and $ denotes (P < 0.05) as compared to vehicle-treated group.

**Suppl Table 4**: Categorization of chromosomal aberrations.

| **Groups** | **% of cells with chromosomal aberrations (CA)** | **Different categories of CA** | | | | |
| --- | --- | --- | --- | --- | --- | --- |
|  |  | **Association** | **Break** | **Fragment** | **Gap** | **Ring** |
| Group 1 | 7.21 ± 0.63 | 0 | 2.99 | 4.57 | 3.44 | 0 |
| Group 2 | 7.14 ± 0.87 | 0 | 2.81 | 2.08 | 2.11 | 0 |
| Group 3 | 41.29 ± 3.48^*^ | 9.02 | 45.56 | 23.75 | 20.08 | 10.59 |
| Group 4 | 32.17 ± 2.96^*^ | 6.89 | 32.20 | 16.48 | 11.51 | 6.92 |
| Group 5 | 24.38 ± 2.57^*^ | 3.81 | 13.98 | 8.11 | 3.83 | 1.27 |
| Group 6 | 18.63 ± 2.74^*^ | 1.18 | 11.37 | 5.90 | 3.55 | 0 |

Data are represented as mean ± SD, n = 6. * denotes (P < 0.001) as compared to the respective control group.

.
